# Innovative Extraction Techniques Using Deep Eutectic Solvents and Analytical Methods for the Isolation and Characterization of Natural Bioactive Compounds from Plant Material

**DOI:** 10.3390/plants9111428

**Published:** 2020-10-24

**Authors:** Milena Ivanović, Maša Islamčević Razboršek, Mitja Kolar

**Affiliations:** 1 Faculty of Chemistry and Chemical Engineering, University of Maribor, Smetanova ulica 17, SI-2000 Maribor, Slovenia; milena.ivanovic@um.si; 2 Faculty of Chemistry and Chemical Technology, University of Ljubljana, Večna Pot 113, SI-1000 Ljubljana, Slovenia

**Keywords:** bioactive compounds, plant material, analytical methods, deep eutectic solvents (DES), natural deep eutectic solvents (NADES), innovative extraction techniques, ultrasound-assisted extraction (UAE), microwave-assisted extraction (MAE)

## Abstract

The growing interest of the food, pharmaceutical and cosmetics industries in naturally occurring bioactive compounds or secondary plant metabolites also leads to a growing demand for the development of new and more effective analysis and isolation techniques. The extraction of bioactive compounds from plant material has always been a challenge, accompanied by increasingly strict control requirements for the final products and a growing interest in environmental protection. However, great efforts have been made in this direction and today a considerable number of innovative extraction techniques have been developed using green, environmentally friendly solvents. These solvents include the deep eutectic solvents (DES) and their natural equivalents, the natural deep eutectic solvents (NADES). Due to their adjustable physical-chemical properties and their green character, it is expected that DES/NADES could be the most widely used solvents in the future, not only in extraction processes but also in other research areas such as catalysis, electrochemistry or organic synthesis. Consequently, this review provided an up-to-date systematic overview of the use of DES/NADES in combination with innovative extraction techniques for the isolation of bioactive compounds from various plant materials. The topicality of the field was confirmed by a detailed search on the platform WoS (Web of Science), which resulted in more than 100 original research papers on DES/NADES for bioactive compounds in the last three years. Besides the isolation of bioactive compounds from plants, different analytical methods are presented and discussed.

## 1. Introduction

Due to the growing social awareness for the consumption of healthy and value-added nutrition, the production of functional foods and various dietary supplements has gained remarkably in importance today and forms a new and fast-growing industrial market worldwide. At the same time, there has also been a growing interest in replacing synthetic additives and colorants with naturally occurring ones. However, not only the food industry is interested in alternative sources of bioactive compounds; the pharmaceutical and cosmetic industries have similar requirements. Secondary plant metabolites that cause pharmacological or toxicological effects in humans and animals are the bioactive compounds from the plants, and some of the most important classes are terpenoids, alkaloids, sulfur-containing compounds, nitrogen-containing compounds and phenolic compounds, which are further divided into several subclasses [1,2]. In between, phenolic compounds have been recognized as excellent supplements in the production of functional foods and as substitutes for the synthetic additives used, due to their numerous beneficial effects on human health. Consequently, many studies have been conducted to optimize their extraction, isolation, separation and determination from natural sources. However, due to their very different and complex structures, there is no universal method adopted for the extraction of all subclasses of plant polyphenols [3].

At the same time, in the various industrial sectors, the value of the products depends not only on the production costs themselves (operating costs per gram of extracted compounds) but also on the production method, i.e., the environmental impact of the processes used [4]. New trends in chemistry and in environmental legislation have placed demands on the use of so-called “green” extraction processes. Indeed, green analytical chemistry (GAC) has evolved from green chemistry, with the challenge of improving the environmental performance of analytical methods. Recently, 12 principles of GAC were proposed by Galuszka et al., 2013 [5]. At the same time, European Union environmental policy and legislation for the period 2010–2050 called for a reduction in the use of petrochemical solvents and volatile organic compounds, as most of them are flammable, volatile and highly toxic [6]. In order to meet all these criteria, so-called “solvent-free” extractions would be the most appropriate; but if all the roles of solvents in the extraction processes are taken into account, there are very few such procedures. So far, the development and production of more environmentally friendly extraction agents is one of the acceptable alternatives. Certainly, water is recognized as the greenest solvent, with the possibility of its use in all industrial sectors, even those strictly limited by legislation, such as the food and pharmaceutical industries. However, the physical and chemical properties of water limit its use in extraction processes. Indeed, water is capable of extracting polar bioactive compounds (even for these compounds, the extractability of water is in the most cases significantly lower compared to organic solvents such as methanol or ethanol), but its extractability is negligible toward to less polar, non-polar and hydrophobic compounds.

Therefore, a class of newly generated fluids, ionic liquids (IL), has received much attention from the scientific community in various research fields since the early 2000s. Although the precise definition of IL is still questionable, they can generally be defined as a mixture of solid compounds with poorly adjusted ions and unique and promising properties that are liquid even at temperatures below 100 °C [7]. The main advantages of IL over traditionally used organic solvents are their negligible vapor pressure, good thermal properties, wide liquid range, wide solubility and miscibility range, suitability for chemical reactions and good recycling properties [7]. However, the studies have shown that the “green” character of IL is at least questionable when considering all the proven shortcomings of these solvents, such as high preparation costs for large-scale applications (in some cases ten times higher than for conventional organic solvents), similar or higher toxicity than organic solvents and low biodegradability [8,9].

Consequently, the new generation of IL known as deep eutectic solvents (DES) or natural deep-eutectic solvents (NADES) has attracted much attention in recent years, with the number of published papers increasing exponentially. More than 60 reviews have described the application of DES/NADES in various research areas, including application in drug discovery and drug delivery systems [10], production of new materials [11], desulfurization of fuel oil [12], application in chromatography [13], application in organic synthesis [14] and others [15]. The selected and cited papers do not include all published reviews based on the DES/NADES, but only the most relevant works. Likewise, their application in extraction processes by various extraction techniques has been described, including ultrasound and microwave assisted extractions [16].

The main objective of this review is to systematically update the results obtained in the last three years with DES/NADES in combination with innovative extraction techniques for the isolation of bioactive compounds from a different plant matrix. Furthermore, analytical methods for the qualitative and quantitative determination of the extracted analytes are discussed. The search on the Web of Science (WoS) resulted in more than 100 original research papers on DES/NADES and bioactive compounds from that period, all of which contained the words “deep eutectic solvents” and “bioactive compounds” or “phenolic compounds” either in the title or in the abstract or in the keywords. Most of these papers were discussed or critically evaluated and are systematically presented in the corresponding tables in the article.

## 2. Analytical Procedures for Determination of Bioactive Compounds from the Plant Material

**Figure 1:**
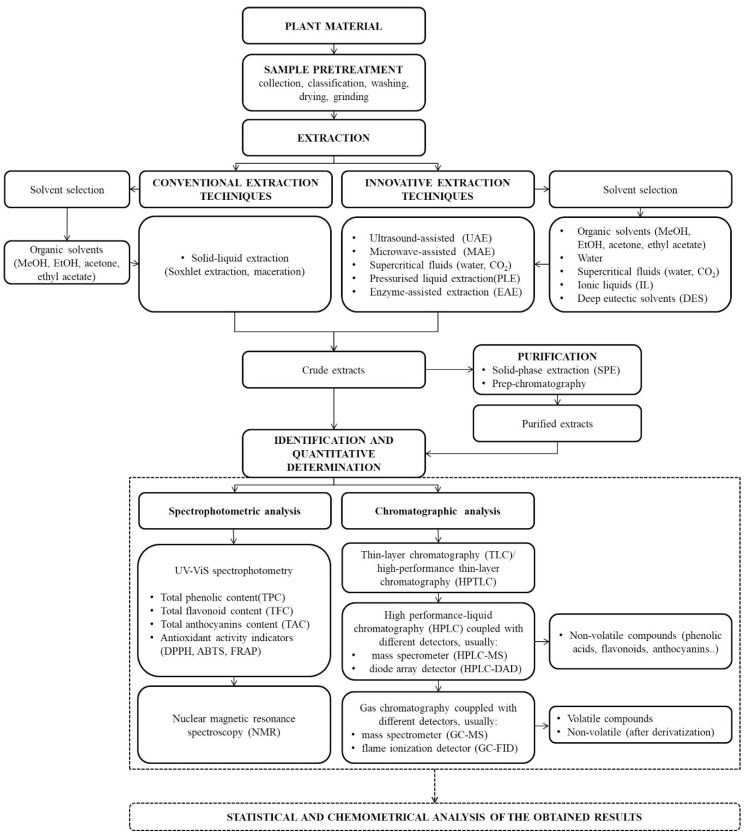
Flow chart of the general analytical protocol for the determination of bioactive compounds from plant material.

In general, the analytical procedure for the determination of bioactive compounds from various natural sources can be divided into several basic steps: sampling, sample pretreatment, extraction of target compounds, purification of crude extracts, qualitative identification and quantitative determination using various instrumental techniques (Figure 1).

The sampling procedure depends strongly on the aims and scopes of the particular investigation, but there are some basic rules applicable to all the analyses. Before the results of a plant analysis could be used effectively, it was necessary to determine which part or portion of the selected plant, vine or tree should be sampled and when sampling should take place [17]. After that, the representative number of plants for sampling and the sampling procedure (random or selected) must be determined [18]. It has also been defined a list with the samples which should be avoided: plants covered with soil, dust, or chemical residues; plants damaged by insects, mechanically injured, or diseased; plants exposed to moisture or temperature and nutritional stress; border-row plants or end-row plants [17,19]. However, with the increasing acquisition of knowledge in the field of bioactive components of plant material and their content as a function of different stress conditions, the process of selecting and sampling representative samples has recently improved. It is known, for example, that the type and content of bioactive components are largely dependent on the geographical location of the cultivated plant, so that plants from the Himalayas can be the source of some unique chemical structures. On the other hand, halophytes that grow on the salty coasts of Louisiana and other states along the Gulf of Mexico may also have unique compounds [20]. Consequently, taking all these findings into account, a detailed recommended protocol for the selection and characterization of plant material for research purposes (in vivo and in vitro studies) was recently published in a review by Kellogg et al. [21]. In addition, sample pretreatment is one of the initial steps of the qualitative and quantitative analysis of bioactive compounds, and pretreatment depends on the matrix of the particular sample. Namely, before the extraction, plant samples are usually treated by milling, grinding and homogenization, which may be preceded by air-drying or freeze-drying [22].

However, extraction is often regarded as one of the most important steps and a critical point in an overall analytical procedure. This step not only helps to remove interferences from the complex matrices, but also to isolate and concentrate the target analytes. The extraction efficiency of an analytical process depends on various factors. In the first place, the extraction technique and the solvents used are the predominant factors, but other variables such as the chemical and physical properties of the target compounds, extraction time, temperature, pH and type of matrix also have an important influence. The main challenge in extraction is to achieve maximum utilization and repeatability of the process in the shortest possible time and with minimum use of energy and chemicals. In general, all extraction techniques used in the isolation of bioactive compounds from plant material can be divided into two main groups: conventional (classical) and modern extraction techniques (Figure 1). Maceration (heating and stirring method) and Soxhlet extraction are the oldest types of extraction techniques and usually uses organic solvents such as methanol (MeOH), ethanol (EtOH), propanol, acetone, ethyl acetate and their mixtures with varying proportions of water [23,24,25]. The extraction solvent, the solid/solvent ratio and the process temperature are the most important parameters when it comes to extraction efficiency [26]. However, these conventional techniques have numerous drawbacks, the most important of which are: long extraction time, high extraction costs (associated with the use of large quantities of organic solvents and energy), non-selective extraction, possibility of degradation/isomerization of analytes due to prolonged heating, negative environmental effects and inappropriate recycling practices for the solvents used [27]. Some of these shortcomings can be eliminated or at least reduced by replacing them with simpler and more effective techniques such as: ultrasound-assisted extraction (UAE), microwave-assisted extraction (MAE), enzyme-assisted extraction (EAE), high intensity pulsed electric field (HIPEF), pressurized liquid extraction (PLE), etc. [28] and/or by the use of green and environmentally friendly solvents such as: water, supercritical fluids, IL and DES/NADES. Since these innovative extraction techniques are actually the subject of this review article, they will be discussed in detail in the following sections.

From the aspects of identification and quantification of extracted bioactive compounds, UV-ViS spectrophotometric methods are the most commonly used due to their simplicity and low costs [29]. The main disadvantages of these methods are the lack of selectivity towards individual bioactive compounds [29] and the possibility to determine only the total content of the targeted bioactive subclass, e.g., total phenolic content (TPC), total flavonoid content (TFC), total anthocyanin content (TAC) and/or total antioxidant capacity expressed as iron (III) reducing capacity (FRAP), 2,2-diphenyl-1-picrylhydrazyl radical assay (DPPH) and 2,2′-azino-bis-(3-ethylbenzothiazolin-6-sulphonic acid) radical assay (ABTS) [30]. However, even in these cases the methods are only selective to a limited extent and are influenced by other compounds with similar physical-chemical properties. On the other hand, nuclear magnetic resonance spectroscopy (NMR) has found an interesting application in the identification of individual bioactive compounds from the complex mixtures. The usefulness of NMR spectrometers is increasingly recognized because of its non-invasiveness, speed rapidity and sensitivity for a large number of compounds in a single measurement and without the need for sample pretreatment [31]. The NMR technique also allows unambiguous identification of chiral isomers [32]. However, due to the high investment and operating costs of the device itself, the justification for the use of NMR technology in the analysis of bioactive compounds can only be found in the structural elucidation of previously unconfirmed compounds. Otherwise, the use of this technique for routine analysis is not economically justified.

**Figure 2:**
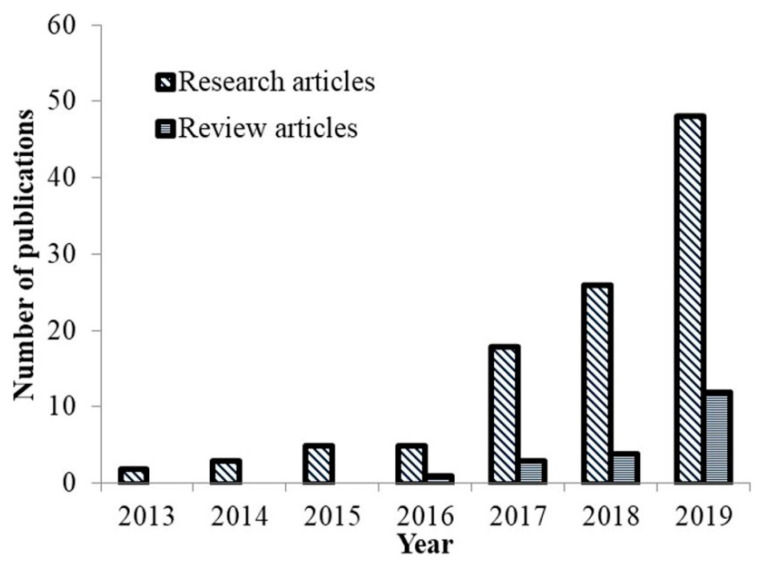
Published works related to the deep eutectic solvents/natural deep eutectic solvents (DES/NADES) in the extraction of bioactive compounds. Web of Science (WoS) search results, all of which contained the words “deep eutectic solvents” and “bioactive compounds” or “extraction” either in the title or in the abstract or in the keywords (*search data*: 18.08.2020.).

Therefore, more powerful and selective techniques, such as: thin-layer chromatography (TLC), liquid chromatography and/or high-performance liquid chromatography (LC/HPLC) and gas chromatography (GC) are required for the identification and quantification of individual bioactive compounds. Traditional TLC is a fast and inexpensive method for the qualitative identification of bioactive compounds in an unknown sample, but the biggest problem in using this method is the impossibility of quantification [33]. With the development of high-performance adsorption layers and sophisticated instrumentation for sample application, chromatogram generation and chromatogram evaluation, high-performance thin-layer chromatography (HPTLC) has become very popular for this purpose [34]. Furthermore, the combination of the powerful separation of TLC/HPTLC with a mass spectrometer (MS) as a detector leads to a unambiguous identification of individual compounds from the different bioactive classes [35,36]. However, the possibility of connecting LC/HPLC devices to different detectors (ultraviolet-visible (UV-ViS), MS, evaporative light scattering detector (ELSD) and electrochemical detector (ECD)), the simplification of sample preparation and a reasonable price of the devices make these techniques the preferred ones for the analysis of bioactive compounds [37]. The choice of the appropriate detector depends on several factors, such as the nature and properties of the analytes being investigated, the sensitivity required and the information to be collected (structural, quantitative, etc.). The most useful detectors in the analysis of bioactive compounds are UV-ViS and MS. UV-ViS is a simple, economical and non-destructive detection method, but it has limited applications that can only be applied to the samples containing compounds that have absorbed radiation at the wavelength of the light source. Another disadvantage of a UV-ViS detector is that it does not provide structural information about the analyte. In the best case it can be used for the determination of subclasses of bioactive compounds, since each family has specific bands of maximum absorption [38]. If the subject of the analysis is a well-known matrix or if the standard compounds are available for structural confirmation of target analytes, the UV-ViS detection will give satisfactory good results. On the other hand, MS is the most selective and powerful detector when it comes to the identification of unknown compounds from the complex extracts (Figure 1 and Figure 2) [39,40].

However, some of the advantages of GC for the analysis of bioactive compounds are difficult to overlook, especially with regard to the separation of compounds, simultaneous analysis and detection limits [41,42]. Likewise, HPLC is not a suitable technique for the quantification of volatile bioactive compounds (e.g., essential oils) and the use of GC is necessary in this case [43].

Nevertheless, taking into account the diversity of bioactive compounds found in different plant material, practical research requires the selection and optimization of each individual step of the entire analytical procedure. Namely, all bioactive compounds in plants can be divided into several main classes according to their biochemical pathways: glycosides, phenolic compounds (phenolic and hydroxycinnamic acids, stilbenes, flavonoids and anthocyanins), tannins, mono-, di- and sequential terpenoids, phenylpropanoids, lignans, resins, alkaloids, furanocoumarins and naphthodianthrones, proteins and peptides [44,45]. All these classes have different chemical structures and physical-chemical properties, which means that different analytical methods are required for their determination. Furthermore, the choice of the appropriate analytical method (especially extraction protocol) depends largely on the matrix itself. For example, bioactive compounds are nowadays extracted from a wide variety of plant matrices, including medicinal plants [46], cereals [47,48], fruits and vegetables [49], edible flowers [50], nuts [51], etc. Of particular importance is certainly the extraction of bioactive compounds from the nus-products of various industries, especially the food industry [52,53,54,55,56].

## 3. Short Historical Overview of Development and Use of DES/NADES

The first groundbreaking study on DES as a possible alternative for IL was reported by Abbott et al. in 2003 [57]. In that paper the authors described the preparation of DES based on different quaternary ammonium salts in combination with ZnCl_2_. Based on their results, DES consisting of choline chloride as ammonium salt, with a melting point of 23–25 °C, was confirmed as the most promising. Since that publication, the number of DES synthesized and characterized has been increasing exponentially. In general, DES can be described as a mixture of two or more solid organic or inorganic compounds which under optimal conditions (temperature and stirring time) liquefies and forms a stable eutectics [58]. According to the results available so far, which are based on various instrumental analyzes (NMR, crystallographic data, fast atomic bomb mass spectrometry and Fourier transform infrared spectroscopy), the DES components are held together by the formation of hydrogen bonds and Van der Waals forces [58,59,60]. Depending on the type of complexing DES agent used, they can be divided into four groups: (I) quaternary ammonium salt and metal chloride; (II) quaternary ammonium salt and metal chloride hydrate; (III) quaternary ammonium salt and hydrogen bond donor and (IV) metal chloride hydrate and hydrogen bond donor [61]. DES type III is most commonly used where choline chloride (hydrogen bond acceptor-HBA) is usually selected as the quaternary ammonium salt, while the typical hydrogen bond donors (HBD) are urea, polyalcohols, sugars, organic acids and phenolic compounds. When the DES forming components contain abundant cellular ingredients (sugars and organic acids) these mixtures are called natural deep eutectic solvents (NADES).

For the production of tailor-made DES/NADES there are three methods that have been adopted and most frequently described: (a) heating and stirring method [60]; evaporation method [60] and freeze-drying method [62]. However, Bajkacz and Jakub described for the first time an ultrasound-assisted protocol for the preparation of 17 different DES, which were additionally used in the extraction processes [63]. The authors postulated the ultrasound-assisted synthesis of DES as the more environmentally friendly and effective method, considering that the synthesis of DES was carried out at the 50 °C and a 15–20 min sonification. Hsieh at al. have also used the ultrasonic waves for the synthesis of polyalcohol-based DES [64]. Based on the results confirmed by Hsieh et al., sonification with a frequency of 40 kHz at room temperature (RT) and a sonification time of up to 5 h should be used to produce clear and colorless liquids [64]. Recently, in a short communication by Gomez et al., a faster, simpler, cheaper and more environmentally friendly method of DES synthesis, based on microwave irradiation was described for the first time [65]. Microwave-assisted technology enabled the authors to generate three different DES, with physical-chemical properties comparable to those produced by conventional method in only 20 s and a significant reduction in total energy consumption. For instance, preparation of DES with the classical heating and stirring method requires a temperature of 80 °C for about 1 h [66,67,68]. Besides, the proposed microwave-assisted technique is still rarely used, probably due to the high initial operating costs.

Interest in DES for the separation processes, in particular the extraction of bioactive compounds, has increased rapidly over the last decade, indicating their great potential in the production of plant extracts for direct use for human consumption. Apart from the proven non-toxicity of these solvents up to a concentration of 600 mM [69], some of the additional advantages for their use in extraction are enriched extraction efficiency, increased stability of target compounds in DES/NADES matrix and unlimited possibilities for their production. One of the first articles described DES for the extraction of various bioactive compounds was published by Dai et al. [60,70]. At the same time, Bi at al. applied the response surface methodology (RSM) for the first time to maximize the extraction yields of two major flavonoids (myricetin and amentoflavone) from *Chamaecyparis obtusa* with polyalcohol-based DES [71]. In addition, Dai et al. have demonstrated an excellent stability of the phenolic compounds of safflower (*Carthamus tinctorius*) in DES compared to 40% MeOH, indicating the possibility of direct use of these extracts in the food and pharmaceutical industry [72]. Since these data, the number of published research articles describing the use of DES/NADES as an extraction medium for the isolation of different classes of bioactive compounds with a broad polarity range has increased significantly (Figure 2). In the year 2016, Espino et al. for the first time reviewed the use of these green solvents in analytical chemistry, taking into account the extraction of phenolic compounds [59].

As already mentioned, in addition to the selection of the suitable solvent, its physico-chemical properties and the extraction conditions applied are decisive for the extraction efficiency. In the case of tailor-made DES/NADES, the different combinations of HBD and HBA used for their production also offer flexible control over the most important physico-chemical properties (viscosity, density, surface tension and pH) [16,53,73]. In general, DES/NADES are highly viscous liquids at room temperature (> 100 cP), which is related to the intensive hydrogen bonding between their constituents [74,75]. Similar patterns were observed for the density of DES/NADES. Based on the results of most authors, DES/NADES are denser than water, with experimentally reported values between 1.04 and 1.63 g cm^−3^ [74,75,76]. The physical-chemical properties of DES/NADES also depend on other factors, such as chemical properties of the starting compounds, molar ratio between the DES/NADES components, temperature and water addition [73]. For example, the viscosity of NADES based on choline chloride and organic acids (organic acids based NADES) is so much higher when oxalic or tartaric acid are used as HBD compared to lactic acid as HBD, even if this applies to the same “class” of NADES. The high viscosity of DES/NADES at RT is in fact one of the main disadvantages of these solvents, which still limits their use in large industrial scale. The paradox lies in the very low evaporation pressure of DES/NADES. From an extraction point of view, this property is preferable, considering that the extraction temperature can be increased without losing the extraction solvents (through evaporation) [77], while on the other hand, a low evaporation pressure is a major disadvantage when it comes to the production of dry extracts. As there are no conditions under which the evaporation of DES/NADES could take place, alternative purification methods should be used.

The viscosities and densities of DES/NADES solvents can be significantly reduced by adding water. However, the addition of water to the DES/NADES system should be done with caution, as a large excess of water could break the hydrogen bonds between the components and thus lose the eutectic properties of the solvent produced [78]. This is probably the reason why researchers have focused on optimizing the addition of water in the extraction process, usually by testing a content between 0% and 70% [68,71,79,80,81]. In most of these studies a water content between 20% and 40% was determined as the optimum. The systematic overview of the interaction between water and DES/NADES is presented in the contributions of El Achkar et al. [73] and Vilkova et al. [78].

From the extraction point of view, the pH value of the solvent is also very important. Hayyan et al. in their study evaluated for the first time the correlation between the pH value and the molar ratio of choline chloride and fructose (1:1; 1.5:1; 2:1; 2.5:1) in the tailor-made DES [82]. Based on their results, a higher molar ratio of fructose, the more acidic solvents are formed. At the same time, they showed that the pH values of the produced solvents were temperature-dependent and ranged between 4.4 and 7.1. All other properties (density, viscosity, surface tension) of the investigated solvents were also temperature-dependent [82]. Furthermore, Skulcova et al. measured pH values of 17 different DES [83]. In that study, choline chloride- and polyalcohol (glycerol or ethylene glycol)- based DES, have demonstrated highest pH values. On the other hand, the lowest pH values were determined for DES based on choline chloride and organic acids, which were between 1.20 and 2.74 at RT and between 0.05 and 2.09 at 60 °C.

It is worth noting that most DES/NADES used for the extraction of bioactive compounds are actually hydrophilic. This is not surprising considering the chemical properties of the target compounds, especially phenolic compounds. However, Křížek et al. have, for the first time, introduced the use of tailor-made hydrophobic DES as extraction media for the isolation of various phytocannabinoids from *Cannabis sativa* [84]. This is a relatively new field of research and only a few papers have been published up to date [85,86]. Since hydrophobic DES components are not miscible with water, an alternative method is required to adjust their physical-chemical properties. In the both published articles, menthol-based DES were used to isolate the most important phytochemicals from the leaves of the *Ginkgo biloba*, and properties of DES was tuned by mixing two or more different hydrophobic solvents [85,86]. Cao et al. have also published an interesting paper describing the simultaneous extraction of hydrophilic and hydrophobic bioactive compounds from the leaves of *Ginkgo biloba* [87]. Thanks to this innovative extraction approach, flavonoids, terpene trilactones, procyanidine and polyprenyl acetates could be extracted in one step. The method was further optimized by RSM, whereby the following conditions proved to be optimal: volume ratio between hydrophilic and hydrophobic DES of 35:5:40 (choline chloride:levunilic acid/choline chloride:malonic acid/methyl trioctyl ammonium chloride:capryl alcohol:octylic acid), the solid/solvent ratio of 1:19.70 (g mL^−1^), the extraction temperature of 65 °C and the extraction time of 41.95 min.

### 3.1. Ultrasound-Assisted DES/NADES Extractions of Bioactive Compounds

Ultrasound-assisted extraction (UAE) is one of the most widely used extraction techniques which is based on the formation of high-frequency (20–1000 kHz) ultrasonic waves that cause cavitation due to the expansion and contraction cycles of the material [88,89,90,91]. The origins of research on the use of the UAE in the extraction of bioactive compounds can be traced back to the 1980s [92], when the technique was described as a methodology with significantly improved extraction yields of target molecules that can be easily performed with standard laboratory equipment (e.g., ultrasonic cleaning bath) [93]. However, the optimization of the UAE method for the respective applications still represents a “challenge” and should take the following factors into account: temperature, extraction time, ultrasound power, ultrasound frequency and the type and volume of solvent used [89]. In general, the positive effect of increasing the temperature during the extraction process is reflected in the reduction of the viscosity and surface tension of the solvents used, resulting in an increase in the overall extraction yield. However, in the UAE, temperatures generally range from RT to 70 °C; higher temperatures are not recommended because of the permissible degradation or isomerization of thermal-sensitive bioactive compounds, which in turn can lead to a reduction in extraction efficiency [94,95,96]. With regard to the extraction time, a compromise should be made between increasing the total extraction yield and reducing the energy input. On the basis of the studied literature, an extraction time of about 30 min was presented by many authors as the optimal [94,97,98]. All advantages and benefits of the UAE technique for the extraction of bioactive compounds have already been described in numerous reviews and are partly listed here [88,89,99,100]. Consequently, the combination of the advantages of UEA with the proven extractability of green DES/NADES for the various bioactive compounds certainly represents a promising alternative to classical long-term extraction protocols.

One of the pioneer’s papers on this topic was published by Cvjetko Bubalo et al., in which the authors demonstrated a possible application of the UAE-DES for the extraction of phenolic compounds from grape skin [101]. In this study, five different DES based on choline chloride and different HBD (glycerol, oxalic acid, malic acid and sorbose) were evaluated and choline chloride:oxalic acid mixture (molar ratio 1:1) with 25% water was selected as the most promising. The predominant phenolic compounds in grape skin are anthocyanins, so the results are not surprising considering the stability of this class of bioactive compounds [101] and the phsyco-chemical properties of the DES studied [83]. In fact, anthocyanins can exist in different forms; at pH 1 (pH value of acidic-based DES) they are mainly present in the form of the red flavylium cation, while at higher pH (between 2 and 4) they are present as blue quinoidal species [101]. From the same reason, the classical extraction protocols for the isolation of pH-sensitive anthocyanin pigments also use acidified organic solvents (MeOH, EtOH or ACN) [102]. The extraction efficiency of the proposed UAE-DES methodology was superior to both, conventional and MAE extraction method. One year later, the same research group published a new paper in which the extraction of anthocyanins from wine lees was described [67]. The methodology was optimized by RSM and the following conditions were found to be optimal: choline chloride:malic acid-based NADES (molar ratio 1:1), UAE extraction time of 30.6 min; ultrasound power of 341.5 W; and water content in NADES of 35.4% (*w*/*w*). The authors also considered for the first time the possibility of transferring the proposed green methodology to industrial processes. They pointed out the following advantages of using NADES compared to conventional extractions: prices of tailor-made NADES comparable to those of traditionally used hazardous organic solvents (the average price is about 2 USD/kg), adjustable physical-chemical properties that allow selective extraction of target molecules, and a truly low vapor pressure of NADES that minimizes the risks of air pollution [67].

In the case of extraction of phenolic acids by the UAE-DES/NADES methods, most authors have found choline chloride:lactic acid-based DES (molar ratio usually 1:2) as the most promising solvent [103,104,105]. The combination of choline chloride with other organic acids such as: *p*-toluenesulfonic acid [66], oxalic acid [106] and citric acid [107] is also interesting.

On the other hand, the combination of DES and UAE for the extraction of different flavonoid subclasses is most optimal when polyalcohol-based DES are used [47,48,108,109,110,111]. For example, Mensur et al. have optimized UAE-DES extraction of flavonoids in common buckwheat sprouts [47]. Nine different DES based on choline chloride and various HBD (acetamide, triethylene glycol, 1,2-propanediol, 1,4-butanediol, urea, ethylene glycpol, glycerol, oxalic acid and malonic acid) were evaluated. Among the DES examined, 80% water solution of choline chloride:ethylene glycol DES was selected as the most promising solvent, and the following conditions were determined as optimal: an extraction temperature of 56 °C and an UAE extraction time of 40 min [47]. Shang et al. were examined 20 different DES with different polarities based on choline chloride, betaine, and L-proline as HBA in combination with different HBD (carboxylic acids, alcohols, sugars and amine) for the extraction of isoflavones from chickpea (*Cicer arietinum* L.) sprouts [48]. Based on the UPLC-QqQ-MS/MS analysis four isoflavones (ononin, sissotrin, formononetin and biochanin A) were identified in all extracts. Choline chloride:propylene glycol in a molar ratio of 1:1 was selected as the best extraction medium, while the other parameters were optimized by RSM: water content in DES of 33%; UAE extraction time of 35 min; extraction temperature of 59 °C and solid/liquid ratio of 40 mg∙mL*^−^*^1^ [48].

In addition, Jeong et al. have proposed a UAE-DES one-step methodology for the simultaneous extraction and characterization of polar bioactive compounds and volatile monoterpenes from peppermint leaves [112]. In this study choline chloride:glucose (molar ratio 5:2) NADES was selected as the most promising solvent. The volatile monoterpenes were analyzed directly with a newly developed and fully optimized method based on headspace solid-phase microextraction coupled to gas chromatography (HS-SPME-GC-MS), while the same extract (without further dilution with organic solvents) was used to determine the main polyphenol indices (TPC and TFC) and antioxidant values (FRAP, DPPH and ABTS). From an economic and environmental point of view, these results seem really promising. However, the proposed method can probably only be used for the chemical characterization of peppermint samples of different origins, while the potential for introduction into large-scale processes is limited.

Besides phenolic compounds, polysaccharides are also one of the important classes of compounds with proven antioxidant, antitumoral, antiviral, anticoagulant and immunostimulant properties [27,113,114]. Polysaccharides are usually extracted from natural sources with hot water. However, based on the authors’ reports, this process of consuming time and energy should be avoided due to an ecological point of view [113]. Zhang et al. have proposed an extraction protocol based on the UAE-DES for the isolation of polysaccharides from *Dioscorea opposite* Thunb (medicinal plant from China). Four DES based on choline chloride and various polyalcohols (glycerol, ethylene glycol, 1,4-butanediol and 1,6-hexanediol) were tested, and this one based on 1,4-butanediol proved to be the most suitable. Similar to the phenolic compounds, a water content of 32.9% (v:v) was determined as optimum, and the other parameters were extraction temperature of 94 °C and the extraction time of 44.74 min [113]. Similarly, Gao et al. evaluated the extraction efficiency of 17 DES for the preliminary extraction of polysaccharides from the seed cake of *Camellia oleifera* Abel [115]. Based on their results, most of the DES tested showed a higher extraction capacity for the polysaccharides compared to extraction in water, while choline chloride:ethylene glycol DES achieved the best result. The crude DES extract was further purified by an aqueous two-phase system [115]. Although the extraction of polysaccharides with DES is undoubtedly a promising alternative in terms of extraction yield and environmental impact, the cost-effectiveness of this process remains questionable.

Furthermore, one of the largest classes of secondary metabolites of microbial, plant and animal origin with an estimated 12,000 molecules are alkaloids. Alkaloids are nitrogenous compounds that are poorly soluble in water but are soluble in organic solvents and can be used in various industries. Takla et al. have for the first time comprehensively compared and evaluated the potential and effectiveness of NADES and non-ionic surfactants for the extraction of amaryllidaceae alkaloids from *Crinum powellii* bulbs [116]. They demonstrated that the extraction was significantly improved compared to traditionally used methods that require the consumption of organic solvents and water. Water-based choline chloride:fructose NADES was selected as an optimal solvent and the other conditions optimized by RSM were a temperature of 50 °C and sonification time of 1 h. In the case of the morphinan alkaloids from *Caulis sinomenii*, NADES based on choline chloride and lactic acid proved to be a best choice [117]. In summary, the number of published research papers describing the use of UAE-DES/NADES for the extraction of various bioactive compounds has increased significantly since 2016. Following the aim of this review paper, Table 1 systematically lists all scientific works published.

### 3.2. Microwave-Assisted DES/NADES Extractions of Bioactive Compounds

Microwave-assisted extraction (MAE), which uses frequencies of 0.3–300 GHz, has recently also been postulated as a novel green approach for the extraction of various bioactive compounds from different natural sources [134,135]. The MAE process can be performed in the “closed-vessel system” or “open-vessel system” [134]. In both cases, the MAE system increases the temperature rapidly, resulting in shorter extraction time and a higher extraction yield of the target analytes. An additional advantage of MAE compared to classical Soxhlet extraction is the possibility to analyze several samples simultaneously. The choice of the appropriate solvent is crucial for maximum process utilization, since many solvents, especially non-polar ones (toluene, hexane and dichloromethane), are transparent to microwave energy [135]. On the other hand, polar solvents such as MeOH, EtOH and ethyl acetate absorb the microwave energy, convert it into heat and thus cause the cell wall to break, and the analytes are transferred into the solvent matrix [136,137]. Taking into account that DES/NADES can efficiently absorb microwave energy [138], it is a rather interesting approach to use DES/NADES as solvent or co-solvent in the MAE for the effective extraction of different classes of bioactive compounds. The extraction efficiency of MAE also depends to a large extent on many other factors, such as the particle size of the sample, the solvent/solid ratio, the applied microwave power, the irradiation time, etc. [135,139]. In this regard, the first research paper describing the use of DES in combination with MAE for the extraction of four major bioactive compounds of *Radix Scutellariae* was presented by Wei et al. [140]. One year later, Chen et al. described the MAE-DES methodology for the isolation of bioactive compounds from the Radix *Salviae miltiorrhizae* [141]. In this study, an optimization of the extraction process was achieved by using an RSM, whereby the authors considered the following extraction conditions as optimal: a temperature of 70 °C, an irradiation time of 11.11 min, a microwave power of 800 W and a solid–liquid ratio of 0.007 g∙mL*^−^*^1^ [141]. They also confirmed a statistically significant increase in extraction yields of five selected analytes compared to the results obtained by hot reflux extraction and UAE. Alañón et al. have used a green and environmentally friendly MAE-NADES methodology for the extraction of 48 different bioactive compounds from olive leaf [80]. After preliminary screening of 11 different NADES, they found choline chloride:ethylene glycol (molar ratio 1:2) to be an excellent solvent, with a statistically significant increase in extraction yield compared to the conventionally used extraction with 80% MeOH. The method was further optimized by RSM and the following extraction conditions were selected as the best: 79.6 °C of temperature, 43.3% of water in NADESs and 16.7 min of irradiation time.

In the recently published paper, Zhao et al. have described a methodology based on MAE-DES pre-treatment in combination with microwave hydrodistillation of essential oils from cumin seed [142]. Based on the results presented, the increased number of compounds was extracted after MAE-DES pre-treatment of the sample, resulting in an increase in the overall extraction yield.

Some of the main disadvantages of the application of MAE in industrial-scale processes are the high operating costs and the high content of impurities in the extracts obtained. In fact, due to the intensive extraction conditions, different analytes could be extracted simultaneously, requiring additional purification steps to produce “ready-to-use” extracts. A limited number of papers based on the use of MAE in combination with DES/NADES for the extraction of bioactive compounds have been published, all of which are systematically listed in Table 2.

### 3.3. New Trends in Extractions of Bioactive Compounds

In addition to the extensively reviewed literature, including the UAE and MAE as the most widely used improved extraction techniques, researchers are working intensively on the development of various other innovative methods. This section therefore systematically describes these efforts, critically examines the main advantages and disadvantages of the proposed methods and disseminates the most promising results.

Chanioti and Tzia have recently compared the influence of different extraction techniques, including homogenization (HAE), MAE, UAE and high hydrostatic pressure-assisted (HHPAE) extraction on the efficiency of the selected DES for the isolation of phenolic compounds from olive pomace [52]. Based on their results, all tested DES showed a superior extraction capacity for the olive polyphenols compared to conventional organic solvents, while the most promising were based on choline chloride and organic acids (lactic acid and citric acid). In addition, HHPAE was selected as the best choice among the extraction processes investigated. The use of HHPAE in the isolation of value-adding compounds from the different matrix is a relatively new and very promising tool. This technique operates at RT or at very low temperatures, leading to results that reduced the percentage of degradation of the thermally sensitive bioactive compounds. At the same time HHPAE reduces extraction time and solvent consumption and improves extraction efficiency in terms of yield, quality and selectivity [150,151]. However, the main disadvantage of HHPAE compared to the other techniques is the high initial investment due to the high cost of the equipment.

Rajha et al. have used a *Ired-Irrad* technology (a now patented infrared-assisted extraction method-IR) in combination with NADES to isolate bioactive compounds from pomegranate peels [152]. According to the results, extracts obtained with the proposed *Ired-Irrad* method showed the highest antioxidant and antibacterial activity compared to extracts obtained with UAE and classical SLE. The IR technology is based on the heating of the plant material by IR radiation which, in combination with a suitable solvent results in selective extraction of the target compounds [152,153]. However, only a limited number of papers on this topic have been published so far, mostly by a single research group, and additional investigation is certainly needed to assess the true potential of this methodology.

The combination of high speed homogenization (HSH) pretreatment with subsequent cavitation-burst extraction (CBE), is also considered to be a promising extraction tool with the potential for use in the extraction of mulberry anthocyanins in combination with NADES [154]. The extraction yield of the proposed method was 25% higher than extraction with conventional organic solvents (acidified ethanol), while the optimal operational conditions were: liquid-solid ratio of 22.4 mL∙g*^−^*^1^; negative pressure of −0.08 MPa and extraction time 30 min with 30% water content in NADES. Even more importantly, acid-based NADES are able to stabilize sensitive anthocyanin molecules [154]. Optimizing the extraction of anthocyanins from natural sources is peculiarly important for the food processing industry, as these compounds have great potential for use as food supplements/coloring additives. Consequently, this extraction method promises to be a safe and stable extraction strategy for obtaining anthocyanins for future applications in pharmaceuticals, food and cosmetics.

Furthermore, Panić et al. have developed an improved microwave-ultrasound combination technique for the extraction of grape-pomace anthocyanins [155]. The special contribution of this work is reflected in the fact that the authors have tested the method developed in the laboratory for the first time on an industrial scale. The total recovery of pure anthocyanins from the NADES extract was about 90%, while the recycling yield of choline chloride:citric acid-based NADES in this system was 78% [155].

The authors have also investigated the influence of vortex agitation on the extraction efficiency of DES for the different bioactive compounds classes [156,157,158]. The main advantages of using vortex-assisted extraction are the low price of the required equipment and the possibility to develop a fast and simple extraction protocol. On the other hand, this method can probably only be used for the characterization process on a laboratory scale, where the requirements for the extract content are low.

Yin et al. for the first time employed tissue-smashing extraction (TSE) with DES to isolate flavonoids from the seeds of *Oroxylum indicum* [159]. The authors highlighted the following advantages of the proposed methodology compared to conventionally applied techniques: lower energy consumption (extraction without heating), ultra-fast extraction speed (total extraction process of 6 min) and increased extraction yield (significantly higher extraction yield compared to extraction with MeOH) [159].

In recent years, supercritical fluid extractions (especially supercritical water extraction-SWE and supercritical CO_2_ extraction) have been recognized as environmentally friendly and cost-effective techniques for the quantitative isolation of different classes of bioactive compounds from natural sources [160]. For the extraction of polar antioxidants, the SWE technique is best suited due to the unique solvent properties of water above boiling point and the high-pressure conditions. However, the major drawback of this extraction technique is the degradation of the thermolabile bioactive compounds at very high extraction temperatures [160]. Consequently, Machmudah et al. have tried to solve this “issue” by adding DES as co-solvent to improve the extraction of xanthone from mangosteen pericarp by SWE [161]. With addition of 30% of DES (alanine:citric acid; molar ratio 1:1), the yield of xanthone was 24.87 mg∙g*^−^*^1^ dried sample, while the TPC was 179.54 mg of gallic acid equivalent (GAE) per g*^−^*^1^ dried sample at extraction temperature of 120 °C in batch system. Such results represented a statistically significant increase compared to extraction under the same conditions without addition of DES [161]. However, caution is required before reaching a final conclusion on the actual improvement of the SWE extraction process by adding DES/NADES as a co-solvent. Namely, as described in the previous sections, excessive water content could break the hydrogen bonds between the DES components and the composite material could lose its eutectic character [78]. The first question that arises is, therefore, whether the increase in the yield of the extraction process is actually due to the influence of the presence of eutectics. In addition, according to the results published so far, it was found that the use of DES/NADES at higher temperatures (which are usually case in SWE) have some limitations with respect to their thermal stability [162,163]. Finally, the purification of the extract in the presence of DES as the co-solvent could be considerably difficult.

### 3.4. Recovering of the Bioactive Compounds from the DES/NADES Extracts

The recovery of the extracted bioactive compounds from the DES/NADES structure continues to be a major challenge in the production of “dried” extracts required for use in the various industrial sectors. Apart from the aim of obtaining dry extract, the importance of solving this problem is also evident in terms of solvent recovery, which would certainly contribute to the overall costs of the entire process. In fact, the “problem” lies in a paradox between extraction efficiency on the one hand and recovery rate on the other. The higher extractability means a deeper interaction between the target compounds and the extraction solvents used, consequently resulting in a difficult separation of the target compounds from the solvent matrix. Furthermore, as mentioned in the previous sections, a low evaporation pressure of the tailor-made DES/NADES makes the work even more difficult. However, some efforts are being made in this area and the most promising results are described in the following paragraphs.

For the recovery of the extracted bioactive compounds, in particular the flavonoids, there are several approaches such as solid phase extraction (SPE) [47,164], macroporous resin adsorption [79,103,111,143,144,148], anti-solvent precipitation [107,164,165], back-extraction [122], and adsorption chromatography [166] that have been evaluated to date.

One of the first attempts to meet this challenge was made by Nam et al., who used water as an anti-solvent to obtain the most important flavonoids (quercetin, kaempferol and isorhamnetin) from *Flos Sophorae*, which where extracted by an optimized extraction method using l-proline:glycerol DES (molar ratio 1:2.5) [164]. However, the results obtained did not meet expectations, while the yield was not more than 50% of the total extracted compounds. In addition, the authors obtained higher recoveries by a simple SPE protocol using the stationary C18 phase, with recoveries for quercetin, kaempferol and isorhamnetin glycosides being 81%, 87% and 87%, respectively.

In 2009, Tian et al. improved a previously described anti-solvent method for the recovery of flavonoids from *Flos Sophorae* extracts and described a new recovery protocol in several subsequent steps [165]. In the first step, the intermolecular forces in the DES were also disrupted by using water as an anti-solvent to reduce the ability to solubilize and interact with extracted phenolic compounds [165]. Subsequently, organometallic framework material (MIL-100 (Cr)) was used as a sorbent for the separation of the target flavonoids from the denatured DES. A critical step in this method was again the recovery of the adsorbed compounds from the MIL-100 (Cr) surface. Namely, due to the highest recoveries of the selected compounds (73%, 89% and 90% for quercetin, kaempferol and isorhamnetin, respectively), the authors proposed elution with MeOH; this in turn led to the use of organic solvents that were avoided during the extraction step.

In addition, Cui et al. have proposed a recovery of the bioactive compounds extracted from *Hippophae Rhamnoides* L. using macroporous resins [143]. In their study, several macroporous resins were tested (NKA-9, AB-8 and D101) for the recovery of flavonoids from the DES crude extract and the resin AB-8 was confirmed as the most promising with an excellent recovery power (more than 72% of the total extract content). In this study a 95% ethanol (v:v) was used as elution solvent, and certainly this stage of the method is more environmentally friendly than the methanol elution described above [165]. The authors also evaluated the possibility of DES recycling and found that the same solvent can be used four times, at least, with a promising result compared to the first extraction cycle (more than 75%). Similarly, Duan et al. have confirmed the resin D101 as the most promising for the recovery of three bioactive flavone-*C*-glycosides (orientine, vitexin and 2″-O-galactopyranosylorientin) from the *Flos Trollii* DES extract [79]. The same microporous risen (D101) and elution with 30% EtOH were used by Cao et al. for the recovery of proanthocyanidin s from *Ginkgo biloba* leaves [157].

Satisfactory good results (with recovery yields of more than 90%) were achieved for the isolation of anthocyanins from the DES/NADES extracts obtained [118,145,155]. For example, Sang et al. were used LX-32 macroporous resin for the separation of total anthocyanins from DES extract of *Lycium ruthenicum* Murr. fruits with a yield of 95% [118]. In the case of anthocyanins from choline chloride:citric acid NADES extract from grape-pomace, Panić et al. used adsorption chromatography on Sepabeads SP207 macroporous resin packed in the glass column [155]. As elution solvent 96% ethanol was used. The recovery of anthocyanins was around 90%, while the recycling yield of choline chloride:citric acid DES in this system was 78%. In addition, the extraction efficiency for grape-pomace anthocyanins of the recycled solvent was 11% lower than that of freshly synthesized NADES.

## 4. Conclusions with Future Perspectives

In recent years, a large number of published articles, only some of which are cited in this paper, indicate the topicality of the field over bioactive compounds. As mentioned above, the interest in this group of chemical compounds has increased considerably when their health benefits became known. As a result, the presence and number of different classes of bioactive compounds in various plant materials including medicinal plants, fruits and vegetables, cereals, leaves of various trees, edible flowers, algae, etc. were determined to date. However, the analysis of bioactive compounds requires a thorough approach. This is because the extraction, sample clean up, identification and quantification processes depend on a number of factors, primarily the chemical properties of the target compounds and the type of sample to be analyzed. Due to the complexity of samples of natural origin, the preparation procedure for the final instrumental determination is a crucial step. Various innovative extraction techniques have been developed so far, and new, more efficient and ecologically acceptable methods are being developed every day. The development of new, green solvents certainly plays an important role in improving analytical methods for the determination of bioactive components. Consequently, from the text described in this review it is evident that the use of DES/NADES in combination with innovative extraction techniques can lead to the improvement in terms of extraction yields of the selected bioactive compounds as well as to the environmental and economic benefits. However, in order to achieve optimal extraction conditions, comprehensive results are required in accordance with the objectives of the individual studies. This is because the extraction capacities of tailor-made DES/NADES are highly dependent on both, the physical-chemical composition of the solvent prepared and the chemical structure of the target compounds. Nevertheless, taking into account all the arguments put forward by the researchers and the works published so far, some general conclusions can be drawn:

1. DES/NADES based on choline chloride as HBA in the combination with different HBD (polyols, organic acids, sugars and amides) are the most commonly used alternative solvents for the extraction of various bioactive compounds from different matrices (Table 1 and Table 2).
2. Sugar-based NADES have the highest viscosity, which makes them difficult to handle and consequently limits their use in the commercial extraction processes [82].
3. Choline chloride:organic acid-based DES/NADES in combination with UAE is the most effective extraction methodology described for the isolation of phenolic acids from the various plant matrices [66,105,106,107,126].
4. In the case of the flavonoid class, the results presented in the majority of the papers have identified choline chloride:polyalchol-based DES/NADES as the most promising solvents (Table 1 and Table 2). However, a complete generalization in this case is not possible. Namely, the flavonoids are additionally divided into several subclasses [167], and the optimization of DES/NADES extraction protocols should be based on the individual subclass.
5. Choline chloride:urea is one of the first synthetized and investigated DES, but this solvent has not found significant application in the extraction of bioactive compounds. One of the few studies in which this solvent has proven to be the best was published by Pal et al. [54]. Indeed, they have confirmed that choline chloride:urea DES is the optimal medium for the isolation of four main flavonoids (quercetin, kaempferol and myricetin) found in the onion skin. However, it should be noted that only three HBD (urea, sucrose and sorbitol) were tested in the present study. This may explain the difference between these results and the others, which were found polyalcohol-based DES to be the most acceptable for isolating the three target flavonoids [110,111,143].
6. Regarding the anthocyanin subclass, the authors found a sugar-based NADES and an organic acid-based NADES as optimal solvents for their isolation from the different matrix [125,130,145,155].
7. In general, the extractability of DES/NADES was in most cases higher compared to conventionally used extraction methods. However, some deviations between the results can be explained by various external factors, such as different origin of the samples analyzed, different growing locations, climatic conditions, time of harvest, storage conditions, etc.

From an industrial point of view, the transfer of the developed methods to scale-up processes is still largely limited. The biggest problems arise in the recovery of target compounds from the extracts obtained and the recycling of the DES/NADES solvents used. Some efforts have been made in this area, but no method has yet been developed that would lead to an absolute elimination of the use of organic solvents, which is certainly one of the fundamental objectives for the use of DES/NADES in extractions. At the same time, however, these protocols have other disadvantages, such as (a) their time-consuming nature and (b) additional costs (cartridges, macroporous resins, absorbents, etc.). One of the possible solutions could also be the direct use of the extracts. In that sense, it is also promising that some studies have shown that the DES/NADES itself has different medical and pharmacological effects (e.g., antibacterial, antifungal, antiviral and anticarcinogenic effects), which would mean that the raw extracts obtained can be used without further purification [10]. However, further research would certainly be necessary, particularly with regard to the toxicity of the tailor-made DES/NADES and their effects on human health. In addition, long-term studies on the stability of the bioactive compounds in these alternative green solvents have also not yet been carried out to the best of our knowledge. One of the few studies that have partially addressed this issue was recently published by Panić et al. [155]. They tested the stability of anthocyanins in different solvents, for 60 days and under different storage conditions (temperatures of −18 °C, 4 °C and 25 °C). The results showed the highest stabilizing capacity of choline chloride:citric acid-based NADES for the selected compounds, with a total degradation of 14% in 60 days, while the degradation in choline chloride:proline:malic acid DES was 70% [155]. In addition, for most industries using bioactive compounds (pharmaceutical, food or cosmetic industries), the organoleptic properties of the final products are extremely important. Therefore, the study of these properties also leaves much space for future research.

Last but not least, we must also look at the economy itself. The first question is whether the use of these solvents (with all the advantages and disadvantages mentioned above) is economically justified. Would the possible transfer of extraction methods based on DES/NADES to industrial sectors require additional investments? In the long term, would their use lead to a shortened lifetime of the extractors and the analytical tools needed for their identification and quantification? All the above questions remain open and certainly offer many possibilities that can be answered in the future [26,168,169].

## Figures and Tables

**Table 1:** Application of DES/NADES in combination with the ultrasound-assisted extraction (UAE) of bioactive compounds from the various plant material in the last three years.

Plant Material	Selected DES/NADES (Number of Tested Solvents)	^∆^Extraction Conditions					Type of Chemical Compounds/Index Determined	Instrumental Analysis	Reference
		A	B	C	D	E			
**Phenolic Compounds**									
Olive cake	Lactic acid:glucose (**3**)	1:5	15	75/1	60	40 °C	**Phenolic acids:***trans*-ferulic acid, caffeic acid **Flavonoids:** tyrosol, 3-hydroxytyrosol, rutin hydrate, apigenin, luteolin	HPLC-DAD	[53]
Onion									
Tomato waste									
Pear waste									
Mulberry leaves (*Morus alba* L.)	^§^ChCl:citric acid (**12**)	2:1	25%	50/1	30	40 °C	**Phenolic acids:** gallic acid, gentisic acid, chlorogenic acid, catechinic acid, vanillic acid, caffeic acid, syringic acid, benzoic aicd **Flavonoids:** epicatechin, rutin, hysperin, astragalin, quercetin	HPLC-UV	[107]
*Lycium ruthenicum* Murr.	ChCl:1,2-propanediol (**4**)	1:2	10%	50/1	45	52 °C	**Anthocyanins:** petunidin-3-*O*-rutinoside-5-*O*-glucoside, malvidin-derivative, petunidin-3-*O*-(glucosyl-*trans*-*p*-coumaroyl)- rutinoside-5-*O*-glucoside, petunidin-3-*O*-(glucosyl-*cis*-*p*-coumaroyl)-rutinoside-5-*O*-glucoside, petunidin-derivative, delphinidin-derivative, petunidin-3-*O*-(caffeoyl)-rutinoside-5-*O*-glucoside, delphinidin-3-*O*-(*p*-coumaroyl)-rutinoside-5-*O*-glucoside, petunidin-3-*O*-(*cis*-*p*-coumaroyl)-rutinoside-5-*O*-glucoside, petunidin-3-*O*-(*trans*-*p*-coumaroyl)-rutinoside-5-*O*-glucoside, petunidin-derivative,malvidin-3-*O*-(*p*-coumaroyl)-rutinoside-5-*O*-glucoside, petunidin-3-*O*-(*p*-coumaroyl)-rutinoside isomer	HPLC-DAD/ESI-MS UPLC-Q-TOF-MS	[118]
Peppermint leaves (*Mentha piperita* L.)	ChCl:glucose (**9**)	5:2	ND	100/1	45	RT	**Phenolic acids:** rosmarinic acid, salvianolic acid **Flavonoids:** eriocitrin, luteolin 7-*O*-rutinoside	UHPLC-Q-TOF-MS	[112]
							TPC, TFC, DPPH, ABTS, FRAP	Spectrophotometry	
*Camellia oleifera* flowers	ChCl:lactic acid (**13**)	1:2	35%	40/1	50	40 °C	**Flavonoids:** quercetin 3-*O*-rhamnoside, kaempferol 3-*O*-rhamnoside, quercetin, kaempferol	HPLC-DAD	[119]
*Pollen Typhae*	ChCl:1,2-propanediol (**8**)	1:4	30%	50/1	35	ND	**Flavonoid aglycons:** quercetin, naringenin, kaempferol, isorhamnetin	HPLC-DAD LC-MS	[110]
*Larrea cuneifolia*	Lactic acid:dextrose (**1**)	5:1	15%	75/1	42	40 °C	**Phenolic acids:** caffeic acid, *trans*-ferulic acid, rosmarinic acid, cinnamic acid, nordihydroguaiaretic acid **Flavonoids:** ( ± ) ctechin hydrate, tyrosol, naringenin, apigenin, quercetin, luteolin, rutin hydrate	HPLC-DAD	[120]
Greek propolis	ChCl:tartaric acid (**1**)	2:1	ND	ND	30	ND	TFC, TCTC, DPPH	Spectrophotometry	[121]
*Lycium barbarum* L.	ChCl:*p*-toluenesulfonic acid (**11**)	1:2	No	50/1	90	25 °C	**Phenolic acids:** chlorogenic acid, ferulic acid, *p*-coumaric acid **Flavonoids:** luteolin, rutin, myricetin, quercitrin, apigenin, hyperoside	HPLC-UV/ViS	[66]
Buckwheat sprouts (*Fagopyrum esculentum* M.)	ChCl:triethylene glycol (**18**)	1:4	20%	20/1	40	56 °C	**Flavonoids:** orientin, isoorientin, vitexin, isovitexin, quercetin-3-*O*-robinobioside, rutin	HPLC-Q-TOF/MS HPLC-PDA	[47]
							TFC	Spectrophotometry	
Safflower (*Carthamus tinctorius* L.)	ChCl:ethylene glycol	1:1	ND	17.8/1	55.9	41 °C	TFC	Spectrophotometry	[111]
Mature citrus (*Nobis tangerine*)	ChCl: levulinic acid:N-methyl urea (**18**)	1:1.2:0.8	20%	20/1	25	50 °C	**Flavonoids:** narirutin, hesperidin, sinensetin, nobiletin, tangeretin, kaempferol-3-*O*-rutinoside, isosinensetin, 3,5,6,7,8,3′,4′-pentamethoxyflavone, 5-hydroxy-6,7,8,3′,4′-penta- methoxyflavon	UHPLC-UV UHPLC-Q-Orbitrap-MS/MS	[122]
Beal (*Aegle marmelos*)	ChCl:oxalic acid (**1**)	1:1	25%	20/1	ND	80 °C	**Phenolic acids:** ascorbic acid, gallic acid, protocatechuic acid, *p*-coumaric acid, ferulic acid **Flavonoids:** quercetin, kaempferol, apigenin	HPLC-UV	[106]
							TPC	Spectrophotometry	
*Byrsonima intermedia* leaves	ChCl:glycerol (**5**)	1:1	20%	70.6/1	25	45 °C	**Phenolic acid:** digalloyl quinic acid Proanthocyanidins: proanthocyanidin dimer, galloylproanthocyanidin dimer, **Flavonoids:** quercetin-*O*-hexoside, galloyl quercetin hexoside, quercetin-*O*-pentoside and galloyl quercetin pentoside	HPLC-MS/MS HPLC-DAD	[109]
							DPPH, ABTS	Spectrophotometry	
Chickpea sprouts (*Cicer arietinum* L.)	ChCl:propilene glycol (**20**)	1:1	33%	40/1	35	59 °C	**Isoflavones:** ononin, sissotrin, formononetin, biochanin A	UPLC-QqQ-MS/MS	[48]
							TFC, DPPH, ABTS	Spectrophotometry	
*Flos Trollii*	ChCL:ZnBr_2_ (**12**)	1:1	48%	23.8/1	28	50 °C	**Flavones:** orientin, vitexin, 2′’-*O*-galactopyranosylorientin	HPLC-UV	[79]
*Epimedium pubescens* Maxim.	ChCl:lactic acid (**18**)	1:2	17.5%	6.4/1	21	25 °C	**Prenylflavonol glycosides:** epimedin A, epimedin B, epimedin C, icariin	HPLC-UV	[123]
Medicus flowers (*Abelmoschus manihot* Linn.)	ChCl:acetic acid (**7**)	1:2	30%	35/1	30	30 °C	**Flavonoids:** hyperoside, isoquercitrin, myricetin	UHPLC-MS/MS	[124]
*Myrothamnus flabellifolia* Welw.	Sucrose:citric acid:water (**4**)	1:1:10	25%	50/1	90	50–55 °C	**Anthocyanins**: cyanidin-3-acetyl glucosamine, cyanidin-3-*p*-coumaryl glucoside, delphinidin-3- glucoside, delphinidin-3-*p*-coumaryl glucoside, malvidin-3-acetyl glucoside, malvidin-3-glucoside, malvidin-3-coumaryl glucoside, pet-3-acetyl glucoside, pet-3-coumaryl glucoside	LC-QTOF-MS/MS	[125]
Rosemary (*Rosmarinus officinalis* L.)	ChCl:1,2-propanediol (**4**)	1:2	10%	52.6/1	120	40 °C	**Phenolic acids:** rosmarinic acid and ferulic acid Flavonoids: 7-methylrosmanol, rutin, naringin Other compounds: caffeine	HPLC-DAD	[126]
							TPC, DPPH, FRAP	Spectrophotometry	
Ginger (*Zingiber officinale Roscoe*)	L-carnitine:1,3-butanediol (**15**)	1:4	25%	30/1	30	50 °C	**Gingerols:** 10-gingerol, 8-gingerol, 6-gingerol	HPLC-DAD	[64]
							FRAP, ABTS	Spectrophotometry	
Elderberry plant (*Sambucus nigra*)	Lactic acid:glycine (**5**)	5:1	15%	16.7/1	28	RT	**Phenolic acids:** neochlorogenic acid, hlorogenic acid, di-caffeoylquinic acid, *p*-coumaroylquinic acid derivative **Flavonols:** quercetin 3-*O*-rutinoside (rutin), quercetin 3-*O*-glucoside (isoquercitrin), isorhamnetin-3-*O*-rutinoside, quercetin	LC-DAD-MS HPLC-DAD	[127]
							TPC, TFC, DPPH, FRAP	Spectrophotometry	
Extra-virgin olive oil	Betaine:glycerol (**10**)	1:2	30%	1/1	20	RT	**Phenolic aclohols:** hydroxytyrosol, tyrosol **Secoiridoid derivatives:** dialdehydic form of oleuropein aglycone, oleuropein aglycone isomer, lygstroside aglycone	HPLC-DAD-ESI-MS HPLC-DAD	[108]
Coffee pulp Cocoa pod husk cocoa husk	ChCl:lactic acid (**6**)	1:2	10%	200/1	3	45 °C	**Phenolic acid:** chlorogenic acid	HPLC-UV UPLC-MS	[105]
							TPC	Spectrophotometry	
*Cortex Fraxini*	Betaine:glycerin (**17**)	1:3	20%	15/1	30	ND	**Coumarins:** aesculetin, aesculin, fraxetin and fraxin	HPLC-UV	[128]
Coffee beans	Betaine:triethylene glycol (**15**)	1:2	30%	66.7/1	20	65 °C	**Phenolic acids:** 3-*O*-caffeoylquinic acid, caffeoylepi-quinic acid, caffeoylepi-quinic acid, 5-*O*-caffeoylquinic acid, 4-*O*-caffeoylquinic acid, 5-pcoumaroylquinic acid, quinolactone, 4-feruloylquinic acid, 3-feruloylquinic acid, quinolactone, 3,4-dicaffeoylquinic acid, 3,5-dicaffeoylquinic acid, 4,5-dicaffeoylquinic acid, caffeoylferuloylquinic acid, caffeoylferuloylquinic acid	HPLC-PDA/ESI-MS	[129]
Chokeberry (*Aronia melanocarpa*)	ChCl:lactic acid (**4**)	1:2	33%	200/1	20	35 °C	**Phenolic acids:** gallic acid, protcatehuic acid, chlorogenic acid, vanillic acid, caffeic acid, syringic acid, *p*-coumaric acid, *trans*-cinnamic acid and ferulic acid **Flavonoids:** epicatechin and quercetin	HPLC-DAD	[130]
							TPC, TFC, TAC	Spectrophotometry	
*Hibiscus sabdariffa*	Citric acid:ethylene glycol (**1**)	1:4	50%	12.5/1	43	ND	TPC, TAC, DPPH, FRAP	Spectrophotometry	[131]
*Moringa oleifera* L. leaves	L-proline:glycerol (**13**)	2:5	37%	12.5/1	15	40 °C	**Phenolic acids:** gallic acid, *p*-hydroxybenzoic acid, rosmarinic acid, **Flavonoids:** ( + )-catechin, vicenin-2, orientin, rutin, hyperoside, kaempferol-3-*O*-rutinoside, isorhamnetin 3-*O*-glucoside, quercetin; apigenin, kaempferol, (−)-epigallocatechin	HPLC-ESI-Q-TOF-MS/MS	[132]
							TPC, TFC, DPPH, ABTS, FRAP	Spectrophotometry	
*Peumus boldus* leaves	L-proline:oxalic acid (**7**)	1:1	20%	10/1	20	RT	21 different phenolic compounds	HPLC-PDA-IT-MS HPLC-ESI-QTOF-MS	[133]
							TPC	Spectrophotometry	
**Alkaloids**									
*Carinum powellii* bulbs	ChCl:fructose (**10**)	5:2	35%	400/1	60	50 °C	Lycorine, crinine, crinamine	HPTLC	[116]
Chinese dark tea (*Camellia Sinensis* L.)	ChCl:lactic acid (**8**)	1:1	31%	34.5/1	38	58 °C	Caffeine	HPLC-UV	[103]
*Caulis inomenii,* *Coptis chinensis,* *Stephania tetrandra, Tetradium ruticarpum, Sophora flavescens*	ChCl:lactic acid (**8**)	1:2	30%	60/1	30	54 °C	Sinomenine, magnoflorine, berberine hydrochloride, epiberberine, coptisine, palmatine hydrochloride, tetrandrine, fangchinolinee, evodiamine, rutaecarpine, matrine, oxymatrine	HPLC-UV	[117]
*Peumus boldus* leaves	L-proline:oxalic acid (**7**)	1:1	20%	10/1	20	RT	Coclaurine, *N*-methylcoclaurine, laurolitsine, isoboldine, boldine, reticuline, isocorydine, laurotetanine, *N*-methyllaurotetanine	HPLC-IT-MS/MS	[133]

^∆^Extraction conditions: A- molar ratio between HBD and HBA in the selected DES/NADES; B-water content in the selected DES/NADES (%); C-solid to liquid ratio (mg∙mL^−1^); D-extraction time (min); E-extraction temperature (°C). ^§^ChCl- choline chloride.

**Table 2:** Application of DES/NADES in combination with the microwave-assisted extraction (MAE) of bioactive compounds from the various plant material in the last three years.

Plant Material	Selected DES/NADES (Number of Tested Solvents)	^∆^Extraction Conditions						Type of Chemical Compounds/Index Determined	Instrumental Analysis	Reference
		A	B	C	D	E	F			
Virgin olive pomace	^§^ChCl:citric acid (**4**)	1:2	20%	80/1	30	60 °C	200	**Secoiridoids:** oleuropein, hydroxytyrsol, **Phenolic acid:** caffeic acid **Phenolic aldehyde:** vanillin **Flavonoids:** rutin, luteolin	HPLC-DAD	[52]
								TPC, DPPH	Spectrophotometry	
Lemon verbena (*Lippia citriodora* L.)	ChCl:lactic acid (**11**)	1:2	32%	100/1	17.1	63.7 °C	700	25 different phenolic compounds including: 8 iridoid glycosides, 12 phenylpropanoid glycosides, 5 flavonoid glycosides	HPLC-DAD-ESI-TOF-MS	[68]
								TPC	Spectrophotometry	
Sea buckthorn leaves (*Hippophae rhamnoides* L.)	ChCl:1,4-butanediol (**12**)	1:3	20%	47.6/1	17	64 °C	600	**Flavonoids:** rutin, quercetin-3-*O*-glucoside, quercetin, kaempferol, isorhamnetin	HPLC-DAD	[143]
*Flos Sophorae Immaturus*	ChCl:1,4-butanediol (**9**)	1:2	25%	38.5/1	20	62 °C	600	**Flavonoids:** rutin, nicotiflorin, narcissin, quercetin, kaempferol, isorhamnetin	HPLC-UV	[144]
Blackcurrant (*Ribes nigrum* L.)	ChCl:lactic acid (**10**)	1:2	20%	76.9/1	14	45 °C	ND	**Anthocyanins:** delphinidin 3-*O*-glucoside, delphinidin 3-*O*-rutinoside, cyanidin 3-*O*-glucoside, cyanidin 3-*O*- rutinoside	HPLC-UV	[145]
								TAC	Spectrophotometry	
*Chuanxiong rhizoma*	ChCl:1,2-propanediol (**20**)	1:1	30%	33.3/1	20	68 °C	1360	**Phenolic acid:** ferulic acid	HPLC-UV	[146]
Onion peel (*Allium cepa* L.)	ChCl:urea (**3**)	1:2	30%	20/1	120	60 °C	850	**Flavonoids:** quercetin, kaempferol, myricetin	LC-MS/MS HPLC-UV	[54]
								TPC, FRAP, DPPH	Spectrophotometry	
Cocoa bean shell	ChCl:oxalic acid (**16**)	1:1	49.4%	50/1	11.4	35.1 °C	800	**Phenolic acids:** gallic acid, caffeic acid **Flavonoids:** catechin, epicatechin	HPLC-DAD	[147]
								DPPH	Spectrophotometry	
Ripe mango (*Mangifera indica* L.)	Sodium acetate: lactic acid (**8**)	1:3	30%	16.7/1	19.7	ND	436	**Xanthone:** mangiferin	HPLC-UV/ViS	[55]
								TPC, FRAP, DPPH	Spectrophotometry	
Mulberry leaves *(Morus alba* L.)	ChCl:glycerol (**12**)	1:2	20%	50/1	18	66 °C	600	**Phenolic acids:** neochlorogenic acid, chlorogenic acid, cryptochlorogenic acid, caffeic acid **Flavonoids:** rutin, isoquercetin, astragalin	HPLC-UV	[148]
Olive leaf (*Olea europa*)	ChCl:ethylene glycole (**9**)	1:2	43.3%	133.3/1	16.7	79.6 °C	ND	48 different phenolic compounds identifed, the most important are: eleoside, elonolic acid, hydroxyoleuropein, luteolin glucoside, oleuropein glucoside, oleuropein, ligstroside	HPLC-DAD-ESI-TOF-MS	[80]
								TPC	Spectrophotometry	
Hibiscus calyces (*Hibiscus sabdariffa* L.*)*	ChCl:oxalic acid (**8**)	1:1	55%	30/1	20	75 °C	700	**Phenolic acids:** chlorogenic acid quinone, neochlorogenic acid, chlorogenic acid, criptochlorogenic acid, coumaroylquinic acid, 5-*O*-Caffeoylshikimic acid **Flavonoids:** myricetin-3-arabinogalactoside, quercetin-3-sambubioside, quercetin-3-rutinoside, kaempferol-3-*O*-sambubioside, quercetin-3-glucoside, methylepigallocatechin, myricetin, quercetin, kaempferol **Anthocyanins:** delphinidin-3-sambubioside, cyanidin-3-sambubioside	HPLC-DAD-ESI-TOF-MS	[149]
								TPC, TAC	Spectrophotometry	

^∆^Extraction conditions: A- molar ratio between HBD and HBA in the selected DES/NADES; B-water content in the selected DES/NADES (%); C-solid to liquid ratio (mg∙mL^−1^); D-extraction time (min); E-extraction temperature (°C); F-microwave power (W). ^§^ChCl- choline chloride. ND- not defined.
